# National assessment of trauma care system capacity in Ecuador: Evaluating institutional resources and practitioner KAPS domains

**DOI:** 10.1371/journal.pgph.0007065

**Published:** 2026-10-26

**Authors:** Doris Sarmiento-Altamirano, Edison Angamarca-Angamarca, Juan Carlos Salamea, Edgar B. Rodas, Luz María Moyano-Vidal

**Affiliations:** 1 Faculty of Public Health and Administration, Universidad Peruana Cayetano Heredia, Lima Perú; 2 Faculty of Medicine, Universidad del Azuay, Cuenca, Ecuador; 3 Faculty of Medicine, Universidad de Especialidades Espíritu Santo, Guayaquil, Ecuador; 4 Department of Surgery, Monte Sinaí General Hospital, Guayaquil, Ecuador; 5 Department of Surgery, Virginia Commonwealth University, Richmond, Virginia, United States of America; 6 School of Human Medicine, Faculty of Health Sciences, Universidad Nacional de Tumbes, Tumbes, Perú; 7 Center for Global Health, Tumbes, Universidad Peruana Cayetano Heredia, Tumbes, Perú; Beth Israel Deaconess Medical Center, UNITED STATES OF AMERICA

## Abstract

We evaluated Ecuador’s trauma care system capacity by analyzing the discrepancy between hospital infrastructure and the individual dimensions of the Knowledge, Attitudes, Practices, and Safety (KAPS) framework among medical specialists. A nationwide, multicomponent cross-sectional study was conducted (2025–2026), comprising a complete structural audit of 49 eligible public hospitals (Levels II and III) and a KAPS survey of 215 trauma-facing specialists representing 36 institutions (73.5% institutional participation rate). Spatial workflow efficiency was assessed via Emergency Department-to-Computed Tomography (ED-CT) transit distances. Independent multivariable Poisson regression models evaluated each KAPS dimension separately, reporting adjusted Incidence Rate Ratios (IRR)accounting for institutional clustering and professional seniority. The institutional audit revealed significant operational constraints: 51.0% of hospitals lacked 24/7 radiological coverage, and 16.3% experienced CT scanner downtime. Spatial ED-to-CT distances were significantly greater in Level III tertiary centers, reaching up to 300 meters (p = 0.006). Among specialists (N = 215 across 36 hospitals), ecological associations with provincial trauma volume yielded wide confidence intervals spanning the null value across domains (Knowledge: rho = 0.113, 95% CI: -0.39–0.61; Reported Practice: rho = -0.160, 95% CI: -0.66–0.34), reflecting low statistical precision from macro-level aggregation. Dimension-specific multivariable models revealed marked inter-specialty divergence: Emergency Physicians exhibited lower Knowledge (aIRR = 0.61; 95% CI: 0.50–0.74), but higher rate ratios in Reported Practices (aIRR = 1.48; 95% CI: 1.34–1.65), Attitudes (aIRR = 1.12; 95% CI: 1.09–1.16), and Safety (aIRR = 1.18; 95% CI: 1.12–1.25) relative to General Surgery. Ecuador’s trauma network exhibits a measurable discrepancy between nominal infrastructure and operational availability. System capacity is constrained by physical architectural barriers and a divergence between practitioners’ subjective perceptions and objective decision-making skills. Strategic priorities must include a unified National Trauma Registry, integrated teleradiology, and continuous scenario-based training.

## Introduction

Injuries represent a predominant cause of global mortality, exerting a disproportionate impact on the economically active population. Annually, trauma arising from unintentional injuries, interpersonal violence, or self-harm accounts for approximately 4.4 million deaths worldwide, constituting 8% of total global mortality [1]. Furthermore, injuries and violence contribute to an estimated10% of years lived with disability (YLDs).The economic burden imposed on national economies is substantial; as healthcare expenditures, productivity losses, and law enforcement costs aggregate into annual losses amounting to billions of dollars [1,2].

In Ecuador, population data analysis from the National Institute of Statistics and Censuses (INEC) from 2022 reveals that interpersonal violence, manifested through homicides and assaults, has become the second leading cause of death nationwide. The national homicide rate experienced a dramatic increase, rising from 6.4 per 100,000 inhabitants in 2015 to 47.3 in 2023. Despite a slight reduction in 2024, the rate remained critically high at 40.4 cases, while trauma-related injuries represent the third leading cause of overall national mortality [3,4].

According to established public health benchmarks, such as the WHO Emergency Care Systems Framework [5] and the WHO Guidelines for Essential Trauma Care [6], effective emergency systems require a synchronized alignment between physical infrastructure and human resource capacity. Previous literature consistently demonstrates that optimizing patient survival and the reducing preventable mortality depend not only on structural capacity (such as equipment, spatial workflows, and diagnostic availability) but also directly /**on strict adherence to evidence-based protocols, coordinated interdisciplinary workflows, and scenario-based decision-making skills among healthcare providers.

Consequently, analyzing clinical outcomes or system preparedness by looking at infrastructure in isolation provides an incomplete diagnostic if it is not coupled with an evaluation of personnel capability. Throughout the acute hospital care continuum, trauma patients require immediate intervention from a diverse array of medical specialists, including emergency physicians, anesthesiologists, general surgeons, and intensivists. Effective interprofessional management the synergistic process by which heterogeneous healthcare providers deliver coordinated care is therefore critical to ensuring timely therapeutic interventions.

However, despite international consensus regarding the necessity of multidisciplinary care, healthcare professionals frequently operate in clinical silos. This fragmentation is driven by several systemic factors, including insufficient mutual understanding of distinct professional roles and competencies, a lack of organizational recognition for interprofessional collaboration, entrenched hierarchical medical cultures, and the widespread absence of standardized clinical protocols [7].

The objective of the present study was to assess national preparedness for severe trauma management in Ecuador by evaluating the discrepancies between hospital structural resources and KAPS domains (Knowledge, Attitudes, Reported Practices, and Safety) among specialists in general surgery, anesthesia, intensive care, and emergency medicine Grounded within international systems frameworks, this study characterizes how physical structural infrastructure aligns with clinicians’ objective knowledge, scenario-based decision-making, and subjective perceptions, establishing the baseline phase for a nationwide educational trauma intervention.

## Methods

### Ethical statement

The study was approved by the Human Research Ethics Committee of the Universidad del Azuay (ID: Exp-082–2025; Resolution: Res-087–2025-CEISH-UDA-RM). Prospective informed consent was obtained from all individual participants via a secure digital platform prior to data collection. This electronic informed consent required participants to read the study objectives, confidentiality terms, and data handling protocols before selecting a mandatory agreement option to access the survey; this digital action served as a documented record of their voluntary authorization. All recruited practitioners were adults over 18 years of age; no minors were included, and parental consent was not applicable.

Institutional structural data were compiled through a field validation of publicly available records (2024 INEC registry) and corroborated via direct observation and professional consultation with active medical staff. Because the assessment focused on physical infrastructure and 24/7 service availability, data that are considered of public interest for healthcare planning, it did not involve access to confidential administrative records or patient-level medical files. The investigation was conducted in accordance with the Declaration of Helsinki to ensure ethical integrity and transparency.

### Study design and population

A multicomponent cross-sectional study was conducted to establish a baseline for a national large-scale educational intervention project (2025–2026). The study followed a quantitative observational, and correlational design to integrate institutional structural data with practitioner profile metrics. This approach enabled a comprehensive assessment of the four dimensions of the Knowledge, Attitudes, Reported Practices, and Safety (KAPS) framework among specialists in general surgery, anesthesia, intensive care, and emergency medicine, alongside the installed capacity for severe trauma management in Ecuador. Reporting follows the Strengthening the Reporting of Observational Studies in Epidemiology (STROBE) guidelines. The recruitment process began in December 2025 with an initial pre-registration phase, followed by the baseline assessment in January and February 2026.

The study employed a dual-level assessment strategy:

1. *Institutional Structural Audit (Phase I):* A comprehensive field assessment was conducted across all 49 public hospitals (Ministry of Public Health-MSP, and Ecuadorian Social Security Institute-IESS) identified as having functional Intensive Care Units (ICUs) achieving 100% national coverage of eligible Level II and III public hospitals. This phase aimed to quantify the country's total secondary and tertiary capacity for trauma response.
2. *Practitioner KAPS Assessment (Phase II):* A nationwide survey was administered to specialized healthcare professionals within these institutions. Although all 49 audited hospitals were invited to participate, a total of 37 institutions agreed to participate and were enrolled in Phase II. To preserve methodological rigor and prevent testing bias, one high-volume trauma center was exclusively allocated for the pilot and semantic validation phase. Consequently, the final analytical clinician cohort was derived from the remaining 36 hospitals (representing a 73.5% effective institutional participation rate).

The study population was selected through nationwide convenience sampling, targeting healthcare professionals who met the following inclusion criteria:

- Active employment in institutions of the Public Comprehensive Health Network (MSP or IESS) equipped with functional Intensive Care Units (ICUs).
- Specialized medical personnel with direct responsibility for the initial management, resuscitation, or surgical management of severe trauma patients (general surgeons, anesthesiologists, emergency physicians, and intensivists).

Exclusion criteria comprised medical residents and healthcare personnel affiliated exclusively with pediatric or obstetric/gynecological hospitals.

### Sampling and coverage

The identification of eligible healthcare facilities was conducted in two stages: a documentary review based on the 2024 INEC registry (43 eligible hospitals with ICUs) [8], followed by field validation that adjusted the final sample to 49 second- and third-level institutions with definitive capacity for severe trauma management.

Across the 36 participating institutions, a baseline universe of N = 256 eligible trauma-facing specialists was identified and invited to participate. From this denominator, a final analytical cohort of n = 215 specialists was successfully recruited, yielding an individual response rate of 84%. Participants were contacted through a multi-stage process coordinated with departments heads (General Surgery, Anesthesia, Emergency Medicine, and Intensive Care) at each hospital. Initial contact was established by telephone to introduce the study objectives, followed by formal invitation to specialized staff. Participation was voluntary, and the survey was completed through a secure online platform. Sample size adequacy was evaluated based on the precision of effect estimates, quantified using 95% Confidence Intervals (95% CI) and standard errors.

The digital survey platform enforced mandatory response fields for all items before submission. Consequently, the dataset was 100% complete with no missing values (0% missingness) across all sociodemographic covariates and the four KAPS outcome dimensions for all n = 215 participants

### Instruments and variables

A structured questionnaire was administered to evaluate three core dimensions:

1. ***Sociodemographic and Occupational Profile*:** Age, gender, medical specialty, prior trauma-specific training and years of active clinical practice.
2. ***Structural Capabilities:*** Assessment of 24/7 availability of critical resources, including operational ICU beds, blood product supply, computed tomography (CT) availability, and on-call specialist coverage (surgeons and radiologists). Furthermore, spatial workflow efficiency was analyzed by measuring the physical distance between the Emergency Department (ED) and the CT scanner suite. A 50-meter threshold was established as the cut-off point for spatial efficiency, serving as a proxy for “zero-transfer” capability during trauma resuscitation [9].
3. ***KAPS Model:*** To ensure structural validity and prevent aggregation bias, the four KAPS dimensions were operationalized using separate scales tailored to their cognitive or psychometric properties:
  - **Knowledge:** Evaluated using five clinical cases based on scenario-based clinical management (score range 0–5). Responses were coded as binary variables (1 = Correct, 0 = Incorrect) based on international trauma guidelines.
  - **Attitudes:** Measured via five items adapted from the Safety Attitudes Questionnaire (SAQ) [8], (score range 0–25). Utilizing 5-point Likert scale (1 = Strongly Disagree to 5 = Strongly Agree), this domain evaluated proactive disposition and clinical prioritization toward core principles Damage Control Resuscitation (DCR).
  - **Reported Practices:** Evaluated the application of the reported application of damage control techniques across five evolving clinical scenarios (score range 0–5). This domain evaluated specialty-specific scenario-driven decision-making pathways and responses were also coded as binary variables (1 = Correct, 0 = Incorrect).
  - **Safety:** Assessed perceptions of workplace safety climates and operational factors (score range: 0–25). Evaluated via a 5-point Likert scale (1 = Strongly Disagree to 5 = Strongly Agree), this subscale integrated validated items from the SAQ [[10,11] adapted to capture human factors and Crew Resource Management (CRM) principles within severe trauma pathways. It operationalized perceptions of teamwork climate, communication barriers, interdisciplinary consensus for damage control triggers, information continuity during clinical transitions, shared mental models for resuscitation targets, and closed-loop verbal communication.

### Validation and pilot testing

The clinical scenario cases used to evaluate the Knowledge and Reported Practices dimensions were developed by the core research team and mapped to evidence-based regional protocols and Latin American for trauma management guidelines to ensure alignment with local epidemiological realities.

To establish construct validity, semantic clarity, and psychometric stability across the four-dimensional KAPS framework, a structured four-stage validation process was executed:

1. Content and Construct Validation by Expert Judgment: An international panel of 12 trauma experts evaluated item relevance and construct representation, yielding an Item Content Validity Index (I-CVI) of 0.83–1.00 and an overall Scale Content Validity Index (S-CVI/Ave) of 0.95.
2. *Metric Validation and Item Calibration by Academic Committee:* An independent committee of 16 university professors audited the domain specifications and scoring rubrics. All 20 items achieved an I-CVI and S-CVI/Ave of 1.00, confirming strict alignment with learning objectives and construct specifications.
3. *Context and Semantic Validation:* To certify language clarity and cultural adaptability, the refined instrument was evaluated by 35 medical residents at Hospital Vicente Corral Moscoso. Item clarity was assessed, leading to the syntactic restructuring of five suboptimal items (I-CVI < 0.78) and consolidating a post-refinement S-CVI/Ave to 0.92.
4. *Pilot Testing, Structural Reliability, and Temporal Stability:* A pilot study was conducted with 20 specialists. Internal consistency was confirmed across all dimensions: dichotomous cognitive/behavioral domains (Knowledge and Reported Practices) yielded a Kuder-Richardson 20, KR-20 = 0.77, while Likert-scale perceptual domains (Attitudes and Safety) evaluated via single-factor Structural Equation Modeling (SEM) yielded a McDonald’s ω = 0.97 (overall Cronbach’s α = 0.81). Two-week test-retest in a subsample (n = 14) demonstrated high temporal stability (ICC = 0.98 for perceptual domains; overall ICC = 0.98).

### Statistical analysis

Data were analyzed using Stata v.19.0. Descriptive statistics summarized participant characteristics, with categorical variables reported as frequencies and percentages, and continuous variables as means (± SD) or medians (IQR). Differences in KAPS scores across medical specialties were evaluated using One-way ANOVA or Kruskal-Wallis tests, followed by Bonferroni post-hoc adjustments for multiple comparisons. Homoscedasticity was verified using Bartlett's test.

The correlation between provincial trauma hospital discharges treated as an aggregate, regional proxy for general system exposure rather than an individual-level clinical volume metric and each of the four independent KAPS dimensions (Knowledge, Attitudes, Reported Practices, and Safety) was evaluated using Spearman’s rank correlation (rho).

To identify independent predictors of each KAPS domain and prevent aggregation bias, four separate multivariable Poisson regression models were constructed, one for each distinct KAPS dimension as an individual dependent variable (Knowledge, Attitudes, Reported Practices, and Safety). Poisson regression models were selected because the KAPS outcome domains represent non-negative, bounded composite scores derived from discrete count items evaluating both objective knowledge items and perceptual domain scales. Furthermore, formal evaluation via the Wolfe-Gould test demonstrated a violation of the proportional odds assumption, indicating that ordinal models were methodologically inadequate.

Modified Poisson regression with robust variance estimators was implemented. Because all KAPS dimensions represent strictly non-negative, right-skewed composite scores, Poisson modeling provides a robust analytical framework that directly yields Incidence Rate Ratios (IRR) without overestimating relative risks. Goodness-of-fit was formally evaluated for each model using residual deviance and Pearson chi-squared tests; overdispersion was ruled out across all outcomes, confirming that standard Poisson distribution assumptions were met, and alternative specifications were not required. Overall model fit for each independent regression was assessed using Log Pseudolikelihood and Pseudo R^2^ metrics.

Results are reported as IRR with corresponding 95% Confidence Intervals (CI). To account for the hierarchical structure of the data (specialists nested within hospitals) and potential intra-group dependency, all four models utilized robust standard errors adjusted for institutional clustering (36 hospitals) via Huber-White sandwich estimators. Covariates included gender, age, years of active clinical practice, medical specialty, prior specialized degrees and trauma-specific continuing medical education (CME). To evaluate the robustness of our Poisson regression findings, sensitivity analyses were performed using multivariable linear regression models with Huber-White cluster-robust standard errors. Because data collection via the secure online platform yielded a complete-case dataset (N = 215) with zero missing data across outcomes and predictors, no imputation procedures (such as multiple imputation) or complete-case sub-analyses were required.

Geospatial analysis was performed to visualize the national provincial distribution of Knowledge (Panel A) and Reported Practice (Panel B) scores. Spatial data were aggregated by province as mean scores and mapped using Datawrapper (datawrapper.de).

## Results

### Participant characteristics and KAPS dimension assessment

A total of 215 specialized medical professionals representing 36 hospitals across 17 provinces participated in the study. Baseline demographics analysis demonstrated no statistical differences among medical specialties regarding age (p = 0.095) or years of active practice (p = 0.054). Significant variations were observed in gender distribution (p = 0.005), geographical allocation across health zones (p = 0.008), and the possession of a prior master’s degree or trauma diploma (p = 0.0012). Continuous medical education (CME) in trauma within the last year also varied significantly by specialty (p = 0.001), where general surgeons reported a participation rate of 43.48% and anesthesiologists reported 9.30%.

Evaluation across the four KAPS dimensions revealed significant differences across specialties. Anesthesiologists and intensive care specialists exhibited higher scores in Knowledge (p < 0.001) compared to the emergency medicine group. Conversely, Emergency physicians presented the lowest knowledge scores alongside the highest median scores in Attitude and Reported Practice (p < 0.001). For the continuous Safety dimension, scores also differed significantly across specialties (p < 0.001) (Table 1).

**Table 1:** Sociodemographic, Occupational, and KAPS Profiles of Participants (n = 215).

Variable	General Surgery (n=92)	Anesthesia (n=43)	Emergency Medicine (n=54)	Intensive Care (n=26)	p-value
**Age (median [IQR])**	41 [37-46]	37 [35-41]	39 [37-43]	42 [36-47]	0.095^a^
**Gender**					
**Male**	60 (65.22)	13 (30.23)	30 (55.56)	18 (69.23)	0.005 ^b^
**Female**	31 (33.70)	30 (69.77)	24 (44.44)	8 (30.77)	
**Other**	1 (1.09)	0 (0.00)	0 (0.00)	0 (0.00)	
**Prior Master’s degree or Trauma Diploma**					
Yes n (%)	17 (18.48)	3 (6.98)	0 (0.00)	0 (0.00)	0.0012^b^
No n (%)	75 (81.52)	40 (93.02)	54 (100.00)	26 (100.00)	
**Years of active practice (median [IQR])**	8 [5-12.5]	7 [4-9]	6 [3-10]	7 [4-13]	0.054 ^a^
**Continuing Medical Education (CME) in trauma (last year)**					
Yes n (%)	40 (43.48)	4 (9.30)	16 (29.63)	6 (23.08)	**0.001^b^**
No n (%)	52 (56.52)	39 (90.70)	38 (70.37)	20 (76.92)	
**Geographical distribution (Health Zones)**					
Zone 1	15 (16.30)	5 (11.63)	7 (12.96)	2 (7.69)	
Zone 2	2 (2.17)	0 (0.00)	0 (0.00)	0 (0.00)	
Zone 3	11 (11.96)	7 (16.28)	10 (18.52)	2 (7.69)	**0.008^c^**
Zone 4	9 (9.78)	1 (2.33)	0 (0.00)	0 (0.00)	
Zone 5	1 (1.09)	0 (0.00)	1 (1.85)	0 (0.00)	
Zone 6	11 (11.96)	12 (27.91)	7 (12.96)	2 (7.69)	
Zone 7	4 (4.35)	6 (13.95)	0 (0.00)	1 (3.85)	
Zone 8	10 (10.87)	3 (6.98)	3 (5.56)	5 (19.23)	
Zone 9	29 (31.52)	9 (20.93)	26 (48.15)	14 (53.85)	
**KAPS Dimensions Median [IQR] or Mean (± SD)**					
Knowledge (/5)	3 [3-4]	4 [3-5]	2 [1-3]	4 [3-5]	**<0.001** ^a^
Attitude (/25)	21 [19-22]	19 [18-21]	23 [21-24]	21.5 [20-25]	**<0.001** ^a^
Reported Practice (/5)	3 [2-4]	3 [2-4]	4 [4-5]	5 [4-5]	**<0.001** ^a^
Safety (/25)	18.16 ± 3.61	19.11 ± 3.19	21.20 ± 2.83	18.96 ± 3.18	**<0.001^d^**

Values are expressed as median [Interquartile Range (IQR)] or mean ± standard deviation (SD), where appropriate. Statistical significance was set at p < 0.05.

^a^Kruskal-Wallis test; ^b^ Chi-square test; ^c^ Fisher’s exact test; ^d^ One-way ANOVA.

### Post-hoc analysis and inter-specialty comparisons

Bonferroni-adjusted post-hoc pairwise comparisons (N = 215) revealed marked dimensional divergence across medical specialties. In Knowledge, Emergency Medicine scored significantly lower than General Surgery (p < 0.001) and Anesthesia (p < 0.001), whereas Intensive Care demonstrated significantly higher scores than Emergency Medicine (p < 0.001). Conversely, Emergency Medicine exhibited significantly higher scores in Attitudes compared to both General Surgery (p < 0.001) and Anesthesia (p < 0.001). A similar pattern emerged in Safety, where Emergency Medicine scored higher than General Surgery (p < 0.001), Anesthesia (p = 0.013), and Intensive Care (p = 0.029). Regarding Reported Practices, both Emergency Medicine (p < 0.001) and Intensive Care (p < 0.001) significantly outperformed General Surgery. Detailed mean differences, standard errors, and 95% confidence intervals for all pairwise comparisons are provided in S1 Table.

### Geospatial distribution of trauma KAPS scores

The geospatial assessment (Fig 1 and Fig 2) revealed statistically significant geographic heterogeneity across provinces for Reported Practice scores across regions, with mean scores ranging from 1.50 to 4.00 out of 5 (Kruskal-Wallis p = 0.004), for Knowledge, provincial means ranged from 1.50 to 3.88 out of 5 (Kruskal-Wallis p = 0.395)When evaluating the relationship between regional trauma volume and clinician KAPS metrics across the 17 provincial units, Spearman rank correlations demonstrated weak to moderate ecological estimates across domains: Knowledge (rho = 0.113; 95% CI: -0.39–0.61; p = 0.666), Reported Practice (rho = -0.160; 95% CI: -0.66–0.34; p = 0.541), Attitudes (rho = 0.247; 95% CI: -0.24–0.73; p = 0.340), or Safety perceptions (rho = 0.164; 95% CI: -0.34–0.66; p = 0.529) (Table 2).

**Table 2:** Correlation between Provincial Trauma Discharge Volume and Mean Knowledge and Reported Practices scores in Ecuador.

Province	Mean Knowledge Scores	Mean Reported Practice Scores	Trauma discharge volume*
Azuay	3.52	3.35	20,600
Bolivar	3	4	2,281
Cañar	3.81	3.9	3,375
Chimborazo	3	3.54	11,349
Cotopaxi	2.66	3.66	6,777
El Oro	1.5	2	15,727
Esmeraldas	2.5	1.5	3,396
Guayas	2.95	2.95	78,528
Imbabura	3.42	3.64	11,021
Loja	3.88	2	11,070
Manabí	3.88	2.88	33,364
Morona Santiago	3.5	3	2,819
Napo	2.5	3	1,991
Pastaza	3.44	2.77	2,419
Pichincha	3.13	3.68	75,848
Sucumbíos	2.78	3.07	1,651
Tungurahua	2.25	1.75	15,329

Trauma discharge volumes reflect consolidated national institutional registries for the 2025 fiscal year. Knowledge and Reported practices score represent the provincial means derived from clinical scenarios (Maximum score = 5 per domain). Spearman's rank correlation (rho) with provincial volume across all four independent domains: Knowledge (rho = 0.113 (95% CI: -0.39 – 0.61; p = 0.666). Reported Practice (rho = -0.160 (95% CI: -0.66 – 0.34; p = 0.541). Attitudes [scale range 0–25] rho = 0.247 (95% CI: -0.24 – 0.73; p = 0.340); and Safety perceptions [scale range 0–25] (rho = 0.164 (95% CI: -0.38 – 0.71; p = 0.529). rho: Spearman's rho.

**Fig 1:**
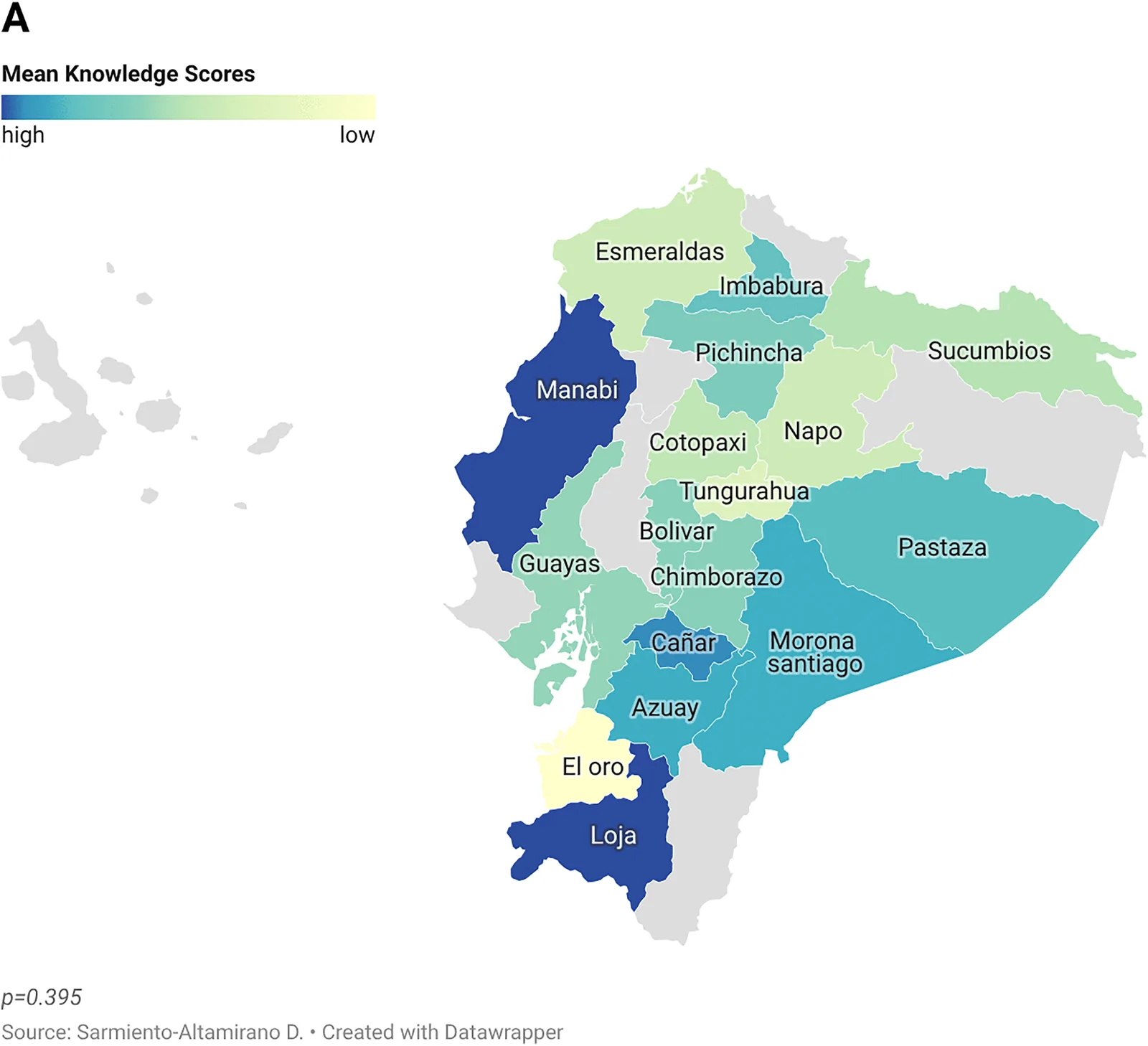
Geospatial distribution of national trauma Knowledge scores across Ecuadorian provinces. Provincial mean Knowledge scores evaluated independently using scenario-based clinical cases (score range: 0–5).

**Fig 2:**
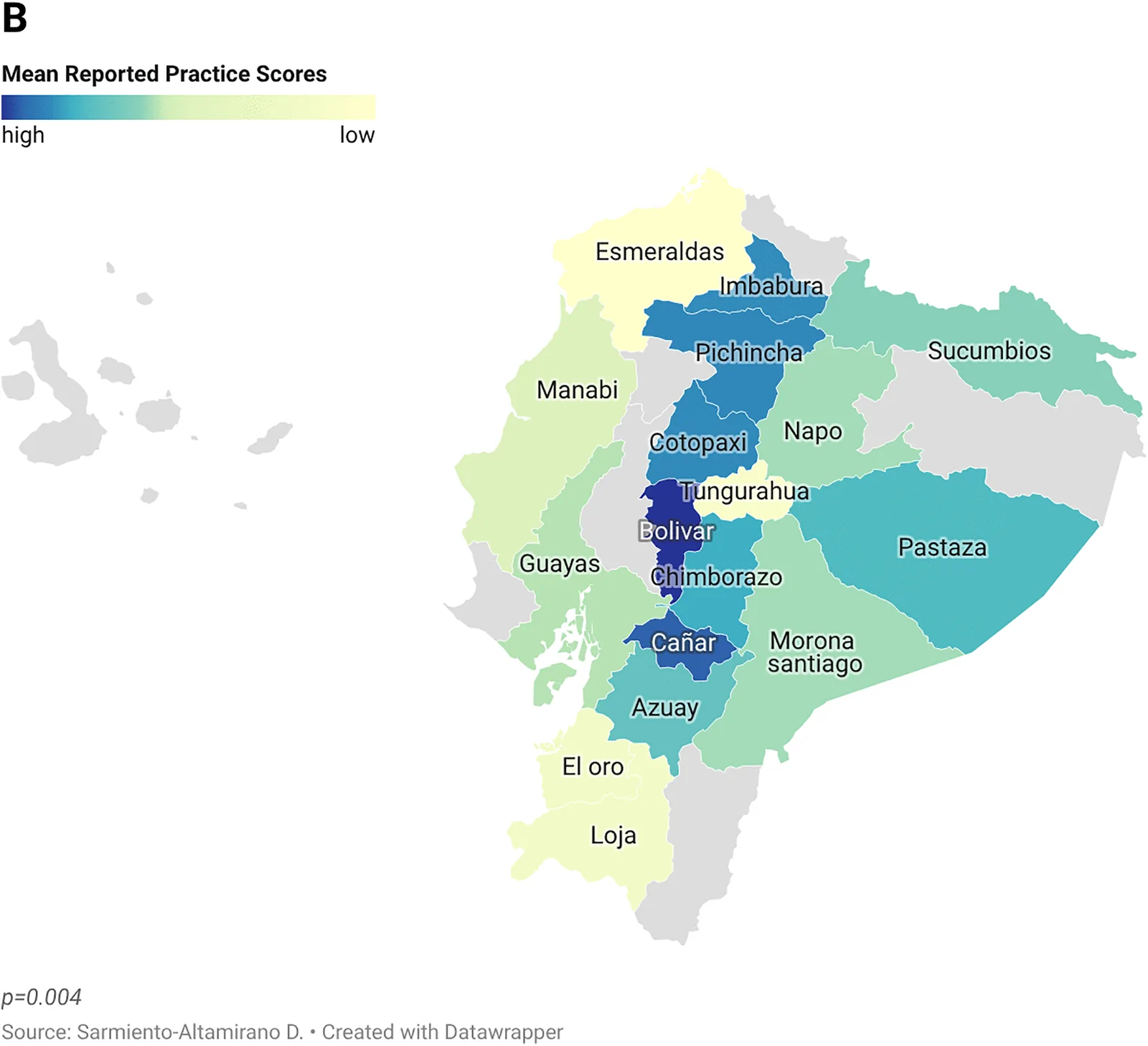
Geospatial distribution of national trauma Reported Practice scores across Ecuadorian provinces. Provincial mean Reported Practice scores evaluated independently using damage control decision-making pathways (score range: 0–5). Base map layer attribution: Visual representation and provincial choropleth maps were independently generated by the authors using Datawrapper (Datawrapper GmbH, Berlin, Germany; map visualization ID: kF3d5 available at https://www.datawrapper.de). Administrative boundary layers are provided via Datawrapper from OpenStreetMap data, © OpenStreetMap contributors, licensed under the Open Data Commons Open Database License (ODbL) (https://www.openstreetmap.org/copyright).

### Multivariable analysis results

Compared to General Surgery (Reference group), Emergency Medicine specialists exhibited lower rate ratios for Knowledge (IRR = 0.61; 95% CI: 0.50–0.74; p < 0.001), but higher rate ratios for Attitudes (IRR = 1.12; 95% CI: 1.09–1.16; p < 0.001), Reported Practices (IRR = 1.48, 95% CI: 1.34–1.65, p < 0.001) and Safety (IRR = 1.18; 95% CI: 1.12–1.25; p < 0.001). Intensive Care specialists demonstrated higher rate ratios for Knowledge (IRR = 1.20; 95% CI: 1.04–1.39; p < 0.05) and Reported Practices (IRR = 1.52; 95% CI: 1.30–1.77; p < 0.001) relative to the reference category. Anesthesiologists showed higher rate ratios for Knowledge (IRR = 1.15; 95% CI: 1.02–1.29; p < 0.05) and Safety (IRR = 1.07; 95% CI: 1.01–1.13; p < 0.001), with no variations observed in the remaining dimensions (Table 3).

**Table 3:** Multivariable Poisson Regression Analysis across KAPS Domains.

Predictor / Model Metric	Knowledge	Attitudes	Reported Practices	Safety
**Medical specialty**	**IRR (CI 95%)**	**IRR (CI 95%)**	**IRR (CI 95%)**	**IRR (CI 95%)**
General Surgery	*Reference*			
Anesthesia	1.15 (1.02–1.29)*	0.97 (0.93–1.02)	1.04 (0.82–1.31)	1.07 (1.01–1.13)*
Emergency Medicine	0.61 (0.50–0.74)*	1.12 (1.09–1.16)*	1.48 (1.34–1.65)*	1.18 (1.12–1.25)*
Intensive Care	1.20 (1.04–1.39)*	1.07 (1.02–1.11)*	1.52 (1.30–1.77)*	1.05 (0.99–1.12)
**Gender**				
Male	*Reference*			
Female	1.02 (0.92–1.13)	0.98 (0.94–1.01)	1.02 (0.94–1.11)	0.99 (0.93–1.05)
Other	0.94 (0.86–1.04)	1.09 (1.04–1.13)*	1.10 (0.99–1.21)	1.11 (1.04–1.17)*
**Age (Years)**	1.00 (0.99–1.01)	1.00 (0.99–1.00)	1.00 (0.99–1.01)	1.00 (1.00–1.01)
**Years of active practice**	1.00 (0.99–1.02)	1.01 (1.00–1.01)	1.00 (0.99–1.02)	1.00 (0.99–1.01)
**Prior Master’s degree or Trauma Diploma**	0.87 (0.76–0.99)*	0.99 (0.93–1.05)	0.79 (0.66–0.94)*	0.97 (0.90–1.04)
**CME in Trauma Last Year (Yes)**	0.90 (0.80–1.01)	0.96 (0.93–1.00)*	1.01 (0.92–1.13)	1.00 (0.94–1.05)
**Number of Observations (n)**	215	215	215	215
**Institutional Clusters (Hospitals)**	36	36	36	36
**Log Pseudolikelihood**	-369.222	-558.796	-369.863	-575.203
**Pseudo R^2^**	0.0525	0.0155	0.0319	0.0153

IRR: Incident Rate Ratio; CI: Confidence Interval. CME = Continuing Medical Education.

Models are fully adjusted for all covariates listed in the table: Medical Specialty, Gender, Age (years), Years of Active Practice, Prior Master's Degree or Trauma Diploma, and CME in Trauma in the Last Year.

Standard errors were adjusted for 36 institutional hospital clusters using robust Huber-White sandwich estimators. All four outcomes represent independent Poisson regression models.*p < 0.05.

To evaluate the robustness of our main findings, a sensitivity analysis was performed using multivariable linear regression models with Huber-White cluster-robust standard errors (S2 Table). The linear models yielded identical directional effects and statistical significance across all four KAPS domains, confirming that the observed specialty-level differences were robust to the choice of modeling framework.

### Institutional capacity and structural resources

Evaluation of 49 hospitals across 21 provinces revealed significant structural heterogeneity regarding required resources for severe trauma management.

1. **Critical Care Infrastructure: Quantitative Bed Fluctuation**

A system-wide comparison between the official 2024 INEC registry and 2026 field validation demonstrated an unstable ICU landscape. Only 30.6% (n = 15) of hospitals maintained a stable bed capacity. The remainder of the network showed high turnover, with an equal proportion of institutions (34.7%, n = 17) reporting a reduction in functional beds and expansion (34.7%, n = 17) (Table 5). This instability suggests that critical care availability is often driven by immediate operational reallocations rather than consistent long-term planning.

2. 24/7 Resource Availability

While basic infrastructure exists, substantial deficits in 24/7 coverage were identified, with marked disparities between providers:

- **Surgical and Transfusion Support:** Most of the evaluated centers demonstrate robust 24/7 operational availability for emergency interventions. 93.8% (n = 46) of hospitals have functional blood banks, and 83.7% (n = 41) maintain on-site surgeons 24/7.
- **Radiology Services:** A critical bottleneck remains as 51% (n = 25) of hospitals lack on-site radiologists for 24/7 coverage. A Chi-squared analysis revealed no significant disparity between providers (p = 0.724), indicating that this deficit is a systemic weakness affecting both MSP and IESS networks.
- **Diagnostic Integrity:** At the time of the survey, 16.3% (n = 8) of Computed Tomography (CT) scanners were non-functional due to maintenance or technical failure, despite being part of formal inventories. These gaps primarily affected MSP centers in the Coastal and Amazon regions.

3. Spatial Efficiency: The Complexity Paradox

The physical distance between the ED and the CT scanner a proxy for diagnostic delay revealed a national median of 40 m [IQR: 15–50 m].

- **Threshold Compliance:** While the median is within the 50-meter clinical benchmark, 40.8% of hospitals exceeded this limit.
- **Statistical Significance:** A Wilcoxon rank-sum test confirmed a significant “Complexity Paradox”: Tertiary-level hospitals (Level III) presented a significantly higher median distance (125 m [IQR: 100–200]) compared to secondary-level hospitals (35 m [IQR: 15–50]) (p = 0.006).
- **Impact:** Notable outliers include Hospital Carlos Andrade Marín (300 m) and Hospital Abel Gilbert Pontón (200 m) (Table 4, Table 5).

**Table 4:** Institutional Capacity and Structural Resources in 49 Public Sector Hospitals.

Province	Hospital		ICU Beds (INEC)2024 (n)	ICU Beds (Survey) 2026 (n)	^*^24h CT	Blood Bank	24h Surgeon	24h Radiologist	ED-to-CT Distance (m)
Azuay	Vicente Corral Moscoso	MSP	13	7	Yes	Yes	Yes	Yes	100
	José Carrasco Arteaga	IESS	21	13	Yes	Yes	Yes	No	10
Bolivar	Alfredo Noboa Montenegro	MSP	0	2	Yes	Yes	Yes	No	15
Carchi	Luis Gabriel Dávila	MSP	6	6	Yes	Yes	Yes	Yes	100
Cañar	Homero C. Crespo	MSP	4	4	Yes	Yes	Yes	No	15
Cotopaxi	General Latacunga	MSP	12	10	Yes	Yes	No	No	20
	Latacunga	IESS	5	4	Yes	No	Yes	No	5
Chimborazo	General Docente de Riobamba	MSP	5	8	Yes	Yes	Yes	No	20
	Riobamba	IESS	6	5	Yes	No	Yes	No	50
El Oro	Teófilo Dávila	MSP	6	6	Yes	Yes	Yes	No	30
	General de Machala	IESS	13	10	No	Yes	Yes	Yes	30
Esmeraldas	Delfina Torres Concha	MSP	20	42	Yes	Yes	Yes	No	50
Guayas	Abel Gilbert Pontón	MSP	32	55	No	Yes	Yes	Yes	200
	Monte Sinaí	MSP	43	24	No	Yes	Yes	No	50
	Dr. Enrique Ortega Moreira	MSP	0	9	Yes	Yes	Yes	No	50
	Guasmo Sur	MSP	6	14	Yes	Yes	Yes	No	50
	Teodoro Maldonado Carbo	IESS	51	45	Yes	Yes	Yes	Yes	100
	Los Ceibos	MSP	29	30	Yes	Yes	Yes	No	15
	Dr. Federico Bolaños Moreira	IESS	6	0	Yes	Yes	Yes	No	8
Imbabura	San Vicente de Paúl	MSP	6	6	No	Yes	Yes	Yes	50
	Ibarra	IESS	6	6	Yes	No	Yes	No	100
Loja	Isidro Ayora	MSP	10	10	Yes	Yes	Yes	Yes	50
	Manuel Ignacio Monteros	IESS	8	8	Yes	Yes	No	No	30
	Martín Icaza	MSP	4	4	Yes	Yes	Yes	No	30
Los Ríos	Babahoyo	IESS	4	9	No	Yes	Yes	No	50
	General Quevedo	IESS	0	5	Yes	Yes	Yes	No	70
Manabí	Verdi Cevallos	MSP	6	6	Yes	Yes	No	No	10
	Miguel Alcívar	MSP	8	6	Yes	Yes	Yes	Yes	50
	Rodríguez Zambrano	MSP	7	4	Yes	Yes	No	Yes	100
	Portoviejo	IESS	0	8	Yes	Yes	Yes	Yes	15
	Napoleón Dávila Córdova	MSP	8	4	Yes	Yes	No	No	50
	Especialidades Portoviejo	MSP	30	25	Yes	Yes	Yes	Yes	150
	Manta	IESS	8	8	Yes	Yes	Yes	No	35
M. Santiago	Macas	MSP	0	3	Yes	Yes	Yes	No	20
Napo	José María Velasco Ibarra	MSP	3	11	Yes	Yes	Yes	No	50
Pastaza	Puyo	MSP	7	5	No	Yes	Yes	No	40
Pichincha	Eugenio Espejo	MSP	23	23	Yes	Yes	Yes	No	100
	Enrique Garces	MSP	8	9	Yes	Yes	Yes	Yes	40
	Pablo Arturo Suárez	MSP	9	10	Yes	Yes	Yes	Yes	7
	Docente de Calderón	MSP	12	12	Yes	Yes	Yes	Yes	100
	Carlos Andrade Marín	IESS	18	35	Yes	Yes	Yes	Yes	300
	San Francisco de Quito	IESS	8	8	Yes	Yes	Yes	No	30
	Quito Sur (HQSur)	IESS	18	19	Yes	Yes	Yes	No	50
Santo Domingo	Gustavo Domínguez	MSP	15	10	Yes	Yes	Yes	No	12
	S. Domingo de los Tsáchilas	IESS	6	6	No	No	Yes	No	10
Santa Elena	Liborio Panchana Sotomayor	MSP	7	6	Yes	Yes	Yes	No	15
Sucumbíos	Marco Vinicio Iza	MSP	0	7	Yes	Yes	Yes	No	50
Tungurahua	Regional de Ambato	MSP	20	15	No	Yes	Yes	No	20
	Ambato	IESS	8	8	Yes	Yes	Yes	Yes	12

*At the time of the survey, several Computed Tomography (CT) scanners were reported as non-functional due to maintenance issues or technical failure, despite being part of the hospital's formal inventory.

**Table 5:** Global Analysis of Institutional Barriers and Structural Efficiency.

Analysis Domain	Comparison/Metric	Statistical Result	Interpretation
Spatial Efficiency	Level III (n = 6) vs. Level II (n = 43)	p = 0.006 (Mann Whitney)	Tertiary centers are significantly less efficient in ED-to-CT distance.
	National Median Distance [IQR]	40 m [15 – 50]	Compliance is high, but 25% of hospitals exceed the critical threshold.
Radiology Coverage	MSP vs. IESS 24/7 Radiologist	p = 0.724 (Chi-squared)	No significant disparity between networks; the deficit is systemic across both.
ICU Stability	Reduction in bed capacity	34.7% (n = 17)	One-third of the network lost functional ICU beds between 2024–2026.
	Expansion in bed capacity	34.7% (n = 17)	High turnover: beds are redistributed rather than increased globally.
	Stability	30.6% (n = 15)	Only 1 in 3 hospitals maintained consistent capacity.

## Discussion

The assessment of the Ecuadorian trauma network reveals a profound decoupling between theoretical hospital complexity and functional operational efficiency. While high-income systems leverage centralization to optimize outcomes [12], our findings reflect the systemic barriers described by Yaqoob et al. (2025), where the availability of tertiary-level infrastructure in low-and middle-income countries (LMICs) does not equate to operational capacity. Just as Yaqoob identified a 60% deficit in portable diagnostics and a lack of specific protocols in Pakistan, our data shows a 51% gap in 24/7 radiology and significant architectural delays, suggesting that physical and operational barriers are systemic hallmarks of fragmented trauma systems [13].

The lack of significant correlation between professional seniority and objective assessment metrics in our study aligns with Beiriger et al. (2026) [14], who demonstrated that career longevity alone is not a predictor of survival. Instead, the literature suggests that survival benefits are primarily driven by individual clinical exposure. However, because our study evaluates macro-level provincial volumes rather than tracked individual-level case logs, these findings constitute an ecological analysis and cannot establish a direct causal link between an individual clinician's specific trauma exposure and their scenario-based decision-making readiness. Within the fragmented Ecuadorian system, high provincial volume may simply reflect regional demand without ensuring that institutional workflows distribute this clinical volume effectively among individual practitioners.

The architectural and professional gaps identified in Ecuador must be interpreted through the “High-Volume Threshold” described by Hatfield et al. (2025) [15]. Their study demonstrates that the survival benefit of surgical volume is exclusive to the top 5% of centers, becoming non-significant for the remaining 95% of the network. In the Ecuadorian context, even the busiest tertiary hospitals may fall below this critical operational threshold or lack the standardized protocols common to elite centers.

This distribution highlights why high regional clinical demands do not automatically aggregate into higher provincial scores across any of the four evaluated KAPS domains. Rather than establishing a true absence of association, the wide confidence intervals observed across provincial correlations reflect the limited statistical precision inherent to macro-level aggregation (n = 17). Due to the ecological nature of this cross-sectional comparison, these data describe regional system characteristics rather than individual learning curves, reinforcing the need for systematic process management rather than relying solely on hospital volume to drive cognitive and scenario-based decision-making skills.

While previous literature, often interprets discrepancies between subjective self-assessments and objective clinical evaluations through frameworks of self-perception bias [16], our findings among specialists are better framed as a dimensional divergence between objective test scores and subjective Likert-scale metrics. In our cohort, high self-assessed perception (Likert scale) contrasts with scores derived from objective Knowledge cases and Practice clinical scenarios.

This observed gap between lower objective knowledge and higher reported practices among emergency medicine specialists may point to differences in assessment formats. Standardized testing formats may favor the structured academic training of anesthesiologists or intensivists over the rapid, algorithmic, and protocol-based decision-making characteristic of ED. Furthermore, emergency physicians operate under a broader clinical scope and varied educational backgrounds, which naturally prioritize immediate stabilization pathways over extensive knowledge.

Although Surapaneni et al. (2026) [17] emphasize that external geospatial access remains the primary hurdle in LMICs, our study reveals that internal architectural barriers are equally critical. The excessive physical distance between the ED and the CT scanner identified in Level III hospitals (p = 0.006) directly contradicts the operational “roadmap” established by Aydin et al. (2025) [18]. According to Aydin, physical proximity and the implementation of teleradiology are essential pillars for mitigating personnel deficits and ensuring the delivery of care within the Golden Hour.

The operational integrity of the system is severely challenged by persistent operational bottlenecks and infrastructure maintenance deficits, similar to those documented in other resource-constrained healthcare systems by Opoku-Gyamfi et al. (2025) [19]. In our cohort the frequent downtime of imaging equipment and the structural absence of standardized clinical protocols transform nominally high-tech facilities into healthcare infrastructure burdened by systemic operational bottlenecks. These frequent diagnostic service disruptions, further exacerbated by the post-pandemic workforce crisis (Phelan et al., 2022) [20], limits the timely delivery of care during the Golden Hour, demonstrating that structural capacity alone does not guarantee functional capability.

The discrepancy between official data and our field observations reveals a reactive “informal adaptation” where Ecuadorian ICUs, being polyvalent, require trauma victims to share resources with general medical and oncological cases. This dilution of specialization underscores the need for integrated, system-level trauma pathways as proposed by Roy et al. (2025) [21]. According to the TRAIN-India consensus, transitioning to inclusive systems, prioritizing low-cost interventions like standardized resource allocation, is essential to address the structural inefficiency identified within the network.

To address these systemic structural inefficiencies, Ecuador must evolve toward a standardized, inclusive National Trauma System. In alignment with international consensus frameworks for LMICs, this transition requires a unified, real-time National Trauma Registry to bridge the data gaps identified in this study. By implementing standardized Quality Indicators (Quality Indicators) and rigorous auditing processes, the healthcare system can ensure that existing infrastructure successfully translates into effective clinical care, moving from nominal institutional capacity to true functional capability.

### Limitations

Structurally, the volatility of the Ecuadorian hospital network and the informal adaptation of general ICUs may hinder the standardization of trauma care. While the institutional interview achieved 100% coverage (49/49 hospitals), the individual clinician survey relied on voluntary participation across 36 of the 49 eligible hospitals (representing a 73.5% effective institutional participation rate). This framework introduces a potential self-selection (volunteer) bias, as specialists with a greater baseline interest in trauma care, more extensive training, or higher perceived familiarity with trauma management protocols may have been more likely to participate.

Furthermore, while objective knowledge and reported practices were evaluated through standardized clinical cases, the attitudes and safety dimensions rely on self-reported perception, which is inherently susceptible to social desirability or response bias. Although our clinician sample (n = 215) represents a highly specialized, trauma-facing cohort across the country's public intensive networks, cautious interpretation is required when extrapolating these findings to lower-complexity facilities or non-specialized personnel.

Additionally, this study links aggregate provincial-level trauma volumes with individual and clustered KAPS scores. This configuration constitutes an ecological analysis; therefore, it is methodologically impossible to determine whether an individual clinician with higher personal trauma exposure possesses superior scenario-based decision-making skills. We explicitly acknowledge the risk of the ecological fallacy, meaning that the observed institutional and provincial-level lack of ecological correlation between volume and KAPS scores does not necessarily apply to individual practitioner sub-dynamics. These findings should be interpreted strictly as an exploratory assessment of systemic fragility in regional trauma surveillance rather than a definitive, generalizable profile of national clinical practice.

## Conclusions

The Ecuadorian trauma network exhibits a measurable discrepancy between institutional infrastructure and operational availability. Regional hospital discharge volumes yielded wide confidence intervals spanning the null value across all four evaluated KAPS domains including knowledge, scenario-driven reported practices, and subjective perception scales, reflecting the limitations of macro-level ecological evaluations. Meanwhile, a marked divergence was observed between practitioners’ objective knowledge scores and their subjective self-assessments of preparedness. Furthermore, the capacity of the network is constrained by documented structural gaps, such as the limited availability of 24/7 radiology coverage.

Addressing these gaps requires a coordinated, multi-level response dividing systemic structural reforms from individual professional responsibilities. Within the realm of the Ministry of Health, regulatory bodies, it is critical to mandate the implementation of a unified national trauma registry alongside scalable, low-cost educational interventions tailored for resource-limited settings. These include simulation-based continuing medical education, regional trauma mentorship networks, and tele-mentoring platforms (such as Project ECHO). Furthermore, educational authorities should transition toward continuous, scenario and protocol based recertification integrated directly into hospital trauma quality improvement programs, rather than relying on career longevity alone.

Conversely, within the realm of medical professionals themselves, there is an immediate responsibility to actively engage in these standardized case-based evaluations, maintain individual alignment with updated protocols, and participate in continuous trauma-specific clinical training. Implementing these distinct institutional and professional actions is recommended to systematically improve the quality and functional availability of trauma care across the healthcare network.

## Supporting information

S1 Table Bonferroni-Adjusted Post-Hoc Pairwise Comparisons Across Medical Specialties at Baseline (N = 215). Pairwise comparisons, standard errors, and confidence intervals across knowledge, attitudes, safety perceptions, and reported practices domains.
(DOCX)

S2 Table Sensitivity analysis: Multivariable linear regression models with cluster-robust standard errors for KAPS baseline scores (n = 215). Linear regression models with Huber-White cluster-robust standard errors accounting for institutional clustering across 36 hospital units at baseline.
(DOCX)

S1 Checklist Inclusivity in global research questionnaire. Completed PLOS questionnaire for inclusivity in global research.
(PDF)
