# Supplementary material for: National assessment of trauma care system capacity in Ecuador: Evaluating institutional resources and practitioner KAPS domains
Source: PLOS Glob Public Health. 2026 Oct 26;6(10):e0007065. doi: 10.1371/journal.pgph.0007065 (PMC13704864; doi:10.1371/journal.pgph.0007065)
Supplement: Supplementary file 1 [file pgph.0007065.s003.pdf]

Inclusivity in global research

PLOS' policy on inclusivity in global research aims to improve transparency in the reporting of research performed outside of researchers' own country or community and ensures that PLOS publications reporting global research adhere to high standards for research ethics and authorship. Authors of relevant research articles may be asked to complete the questionnaire below, which outlines ethical, cultural, and scientific considerations specific to inclusivity in global research. This questionnaire may be requested when researchers have travelled to a different country to conduct research, if research uses samples collected in another country, research with Indigenous populations or their lands, or if research is on cultural artefacts. Researchers travelling to another country solely to use laboratory equipment will not normally be required to complete the questionnaire. However, the questionnaire can be requested at the journal's discretion for any submission – if you have been requested to complete this questionnaire by the PLOS journal you submitted to, please do so.

Please complete the questionnaire below and include this as a Supporting Information file with your manuscript. Note that if your paper is accepted for publication, this checklist will be published with your article in the supporting information files. Please ensure that you reference the checklist in the main body of your manuscript. We suggest adding a subsection 'Inclusivity in global research' to your Methods section and adding the following sentence: "Additional information regarding the ethical, cultural, and scientific considerations specific to inclusivity in global research is included in the Supporting Information (SX Checklist)"

The questions have been designed to be applicable to a wide range of study types, and there are subsections for both human subjects research and non-human subjects research. If any of the questions are not relevant to your research please mark them as "N/A" as appropriate.

Ethical considerations, permits and authorship

This section is applicable to all research types.

Provide details as to who granted permissions and/or consent for the study to take place in the Methods section of your manuscript. This should include the names of **all** ethics boards, governmental organizations, community leaders or other bodies that provided approval for the study. If individuals provided approval refer to these people by their role or title but do not list their name(s).

Reported on page number: 3

If there were any deviations from the study protocol after approval was obtained please provide details of these changes in the Methods section of your manuscript.

Reported on page number: N/A

Did this study involve local collaborators that are residents of the country where the research was conducted or members of the community studied? If you do not have any authors from said communities, please provide an explanation for this below.

Yes. The investigation was fundamentally led, designed, and executed by local researchers and clinical practitioners currently residing in Ecuador.
Dr. Doris Sarmiento-Altamirano (Principal and Corresponding Author) and Dr. Juan Carlos Salamea are academic and medical professionals permanently based in Cuenca, Ecuador, representing the *Universidad del Azuay*.
Dr. Edison Angamarca-Angamarca is an active surgeon in the public health system and an academic investigator based in Guayaquil, Ecuador (Universidad de Especialidades Espíritu Santo and Monte Sinaí General Hospital).
Dr. Edgar B. Rodas (Virginia Commonwealth University, USA) is an expert Ecuadorian trauma surgeon who maintained close collaborative engagement with the national surgical field despite his current US affiliation.
Dr. Luz María Moyano-Vidal (Universidad Peruana Cayetano Heredia / Universidad Nacional de Tumbes, Perú) serves as the primary doctoral research advisor, providing formal academic and methodological supervision within the framework of a regional collaborative PhD track.
Thus, the entire data collection, institutional field validation, and target cohort engagement were directly conducted by the local authors rooted within the Ecuadorian medical system, ensuring complete structural integration.

Everyone listed as an author should meet PLOS' criteria for authorship and all individuals who meet these criteria should be included in the author byline, rather than the acknowledgements. For further information please see the journal's Authorship Policy.

Human subjects research (e.g. health research, medical research, cross-cultural psychology)

Did you obtain written informed consent from a representative of the local community or region before the research took place? How did you establish who speaks for the community? Details of written informed consent obtained from study participants should be reported separately in the Methods section of your manuscript.

For this national assessment, institutional structural indicators were primarily retrieved from open-access public platforms and official national repositories (such as the National Institute of Statistics and Censuses - INEC), which host public-interest healthcare planning data. These baseline data were directly corroborated through the study participants (active medical specialists) during the field validation phase. Therefore, formal administrative permission from individual hospital directors was not required.
Regarding individual participants, prospective electronic informed consent was obtained from all participating medical specialists through a secure digital platform prior to data collection. This protocol required participants to read the complete study objectives, confidentiality guarantees, and data handling terms before clicking a mandatory agreement button to access the survey. This electronic action served as a documented record of their voluntary authorization. Full details are reported in the "Ethics Statement" subsection at the beginning of the Methods section.

How did members of the local community provide input on the aims of the research investigation, its methodology, and its anticipated outcome(s)?

Done. We have expanded and clarified the Validation and Pilot Testing subsection. The five simulated scenarios were mapped to standard international ATLS guidelines and validated by 12 regional trauma experts (S-CVI/Ave: 0.95–1.00). Furthermore, to guarantee contextual face validity and evaluate operational feasibility, the tool was formally piloted at the Hospital Vicente Corral Moscoso (a high-volume, reference trauma center in Ecuador) with 35 residents and 20 medical specialists. Items with poor semantic performance (I-CVI < 0.78) were adjusted, ensuring a robust post-refinement scale structure.

When engaging with the local community, how did you ensure that the informed consent documents and other materials could be understood by local stakeholders?

All informed consent documents, clinical case scenarios, and evaluation questions were natively developed, structured, and administered in Spanish, the primary language of all national stakeholders and participating practitioners. Furthermore, during the semantic validation phase at the Hospital Vicente Corral Moscoso, the phrasing, technical vocabulary, and digital layout were thoroughly tested with local residents and specialists. This process allowed us to adjust items with suboptimal readability and ensure that all materials were perfectly clear, unambiguous, and contextually aligned with the everyday clinical terminology used within the Ecuadorian public healthcare network.

Will the findings of the research be made available in an understandable format to stakeholders in the community where the study was conducted (e.g. via a presentation, summary report, copies of publications, etc.)? Please provide details of how this will be achieved.

Yes. The findings of this research will be actively disseminated to local and national stakeholders through a multi-channel strategy. First, copies of the peer-reviewed publications resulting from this study will be shared directly with the medical directorates and academic boards of the participating hospitals (including the Hospital Vicente Corral Moscoso) to provide them with an objective diagnosis of their trauma care capacity. Second, an executive summary report written in Spanish will be prepared and delivered to local health planning authorities and academic institutions, such as the Universidad del Azuay, to support medical curriculum design and resource allocation strategies. Finally, the main conclusions will be presented at national surgical and public health congresses to foster discussion among the wider Ecuadorian medical community.

Non-human subjects research using specimens/ animals collected as part of the study, or those housed in archival collections. Examples include archaeology, paleontology, botany and zoology.

Did the permission you obtained from a local authority to perform the study include an agreement on access to outputs and benefit sharing? This may include procedures to enable fair distribution of the benefits and resources arising from the research performed. Please include any details of Prior Informed Consent and Benefit Sharing Agreements obtained. These may be required by field-specific regulations, for example the Convention on Biological Diversity (CBD) and the associated Nagoya Protocol.

N/A

If the material used in your study was imported, please A) provide the year it was imported and B) indicate whether permits were obtained to import/export the materials used, C) provide details of any permits obtained. If this information is not available, please indicate this.

N/A

If you used archival specimens, please state how the material used in your study was acquired by the institute it is held in and provide details of any permits obtained for the original excavations/ sample collection. If this information is not available, please indicate this.

N/A

How was the potential cultural significance of the materials collected in your study to local communities considered in your research design? Were Indigenous peoples and/or local researchers and institutions involved with archaeological excavations / collection of specimens? If so, please provide a description of their involvement.

N/A

If your manuscript includes photographs of human remains please indicate whether authors obtained permission from descendants or affiliated cultural communities to do so.

N/A
