# Supplementary material for: National assessment of trauma care system capacity in Ecuador: Evaluating institutional resources and practitioner KAPS domains
Source: PLOS Glob Public Health. 2026 Oct 26;6(10):e0007065. doi: 10.1371/journal.pgph.0007065 (PMC13704864; doi:10.1371/journal.pgph.0007065)
Supplement: Supplementary file 2 [file pgph.0007065.s001.docx]

#### S1 Table. Bonferroni-Adjusted Post-Hoc Pairwise Comparisons Across Medical Specialties at Baseline (N = 215)

| **KAPS Outcome Domain** | **Pairwise Specialty Comparison** | **Mean Difference (MD)** | **Std. Error** | **Bonferroni p-value** | **95% CI** |
| --- | --- | --- | --- | --- | --- |
| **Knowledge** | Anesthesia vs. General Surgery | 0.31 | 0.22 | 0.913 | [-0.26, 0.88] |
|  | Emergency Medicine vs. General Surgery | -1.43 | 0.20 | < 0.001* | [-1.97, -0.90] |
|  | Intensive Care vs. General Surgery | 0.49 | 0.26 | 0.364 | [-0.20, 1.18] |
|  | Emergency Medicine vs. Anesthesia | -1.74 | 0.24 | < 0.001* | [-2.38, -1.11] |
|  | Intensive Care vs. Anesthesia | 0.18 | 0.29 | 1.000 | [-0.59, 0.95] |
|  | Intensive Care vs. Emergency Medicine | 1.92 | 0.28 | < 0.001* | [1.18, 2.66] |
| **Reported Practices** | Anesthesia vs. General Surgery | 0.04 | 0.21 | 1.000 | [-0.52, 0.59] |
|  | Emergency Medicine vs. General Surgery | 1.17 | 0.19 | < 0.001* | [0.65, 1.68] |
|  | Intensive Care vs. General Surgery | 1.28 | 0.25 | < 0.001* | [0.62, 1.95] |
|  | Emergency Medicine vs. Anesthesia | 1.13 | 0.23 | < 0.001* | [0.52, 1.74] |
|  | Intensive Care vs. Anesthesia | 1.25 | 0.28 | < 0.001* | [0.50, 1.99] |
|  | Intensive Care vs. Emergency Medicine | 0.12 | 0.27 | 1.000 | [-0.60, 0.83] |
| **Attitudes** | Anesthesia vs. General Surgery | -1.12 | 0.49 | 0.137 | [-2.42, 0.18] |
|  | Emergency Medicine vs. General Surgery | 2.14 | 0.45 | < 0.001* | [0.94, 3.35] |
|  | Intensive Care vs. General Surgery | 1.11 | 0.59 | 0.360 | [-0.45, 2.67] |
|  | Emergency Medicine vs. Anesthesia | 3.26 | 0.54 | < 0.001* | [1.82, 4.70] |
|  | Intensive Care vs. Anesthesia | 2.23 | 0.66 | 0.005* | [0.48, 3.98] |
|  | Intensive Care vs. Emergency Medicine | -1.03 | 0.63 | 0.614 | [-2.71, 0.64] |
| **Safety** | Anesthesia vs. General Surgery | 0.95 | 0.61 | 0.714 | [-0.67, 2.58] |
|  | Emergency Medicine vs. General Surgery | 3.04 | 0.57 | < 0.001* | [1.54, 4.55] |
|  | Intensive Care vs. General Surgery | 0.80 | 0.73 | 1.000 | [-1.15, 2.75] |
|  | Emergency Medicine vs. Anesthesia | 2.09 | 0.67 | 0.013* | [0.29, 3.88] |
|  | Intensive Care vs. Anesthesia | -0.15 | 0.82 | 1.000 | [-2.34, 2.03] |
|  | Intensive Care vs. Emergency Medicine | -2.24 | 0.79 | 0.029* | [-4.34, -0.15] |

*Post-hoc pairwise comparisons derived from one-way ANOVA with Bonferroni adjustment for multiple hypothesis testing. *p < 0.05*
