# Supplementary material for: National assessment of trauma care system capacity in Ecuador: Evaluating institutional resources and practitioner KAPS domains
Source: PLOS Glob Public Health. 2026 Oct 26;6(10):e0007065. doi: 10.1371/journal.pgph.0007065 (PMC13704864; doi:10.1371/journal.pgph.0007065)
Supplement: Supplementary file 3 [file pgph.0007065.s002.docx]

**S2 Table. Sensitivity analysis: Multivariable linear regression models with cluster-robust standard errors for KAPS baseline scores (n = 215)**

| **Predictor / Covariate** | **Knowledge β (95% CI)** | **Reported Practices β (95% CI)** | **Attitudes β (95% CI)** | **Safety Perception β (95% CI)** |
| --- | --- | --- | --- | --- |
| **Medical Specialty** |  |  |  |  |
| *General Surgery* | *Reference* | | | |
| *Anesthesiology* | +0.49 (0.06, 0.92)* | +0.12 (-0.60, 0.83) | -0.61 (-1.69, 0.48) | +1.26 (0.11, 2.41) |
| *Emergency Medicine* | -1.27 (-1.73, -0.81)* | +1.32 (0.98, 1.66)* | +2.41 (1.67, 3.15) | +3.28 (2.15, 4.40) |
| *Intensive Care* | +0.65 (0.07, 1.24)* | +1.42 (0.80, 2.03)* | +1.33 (0.23, 2.44) | +0.92 (-0.35, 2.19) |
| ***Age (years)*** | +0.004 (-0.03, 0.04) | +0.003 (-0.04, 0.05) | +0.02 (-0.03, 0.08) | +0.04 (-0.04, 0.11) |
| ***Years of active practice*** | +0.01 (-0.04, 0.07) | +0.02(-0.05, 0.08) |  |  |
| ***Gender*** |  |  |  |  |
| *Male* | *Reference* | | | |
| *Female* | +0.07 (-0.28, 0.41) | +0.08 (-0.21, 0.36) | -0.49 (-1.22, 0.25) | -0.24 (-1.56, 1.08) |
| *Other* | -0.18 (-0.52, 0.16) | +0.27\ (-0.04, 0.57) | +1.75 (0.91, 2.60)* | +1.90 (0.78, 3.03)* |
| ***Prior Master’s degree or Trauma Diploma*** | -0.54 (-1.06, -0.02)* | -0.73 (-1.34, -0.13)* | -0.20 (-1.46, 1.06) | -0.57 (-2.01, 0.87) |
| ***CME in last year*** | -0.32 (-0.72, 0.08) | +0.06 (-0.31, 0.42) | -0.77 (-1.54, -0.00)* | -0.09 (-1.19, 1.02) |

Linear regression models with Huber-White cluster-robust standard errors accounting for institutional clustering across 36 hospital units at baseline (n = 215). All models are fully adjusted for Medical Specialty, Age, Years of Active Practice, Gender, Prior Master's/Fellowship, and CME in Trauma in the last year. β: Unstandardized regression coefficient (mean score difference relative to General Surgery). *p < 0.05
